# Multifunctional Stimuli-Responsive Polyaniline-Based Conductive Composite Film

**DOI:** 10.3390/polym17060759

**Published:** 2025-03-13

**Authors:** Wenxin Wang, Huiting Cheng, Xiaobin Zhang, Huan Yang, Haoxiang Ma, Zhiwen Wang, Yijun Chen, Xin Chen, Yihan Pu, Yijun Shen, Qi Chen

**Affiliations:** 1 State Key Laboratory of Tropic Ocean Engineering Materials and Materials Evaluation, Collaborative Innovation Center of Marine Science and Technology, School of Marine Science and Engineering, Hainan University, Haikou 570228, China; wenxinwang@hainanu.edu.cn (W.W.);; 2 School of Materials Science and Engineering, Hainan University, Haikou 570228, China; 3 Deep Sea Engineering Division, Institute of Deep Sea Science and Engineering, Chinese Academy of Sciences, Sanya 572000, China; 4 School of Chemistry and Chemical Engineering, Hainan University, Haikou 570228, China

**Keywords:** shape memory polymer, conductive film, polyaniline, stimuli-responsive, electrical actuation, multifunctional composite

## Abstract

There is a growing demand for multifunctional materials that can meet the increasingly complex needs of modern society. The combination of functionalization and intellectualization promotes the development of multifunctional smart materials. These materials are not only required to possess excellent basic properties, but also need to integrate multiple functions to adapt to various application scenarios. In this study, a simple solution co-blending method for preparing a polyaniline-based multifunctional conductive composite film was proposed. This methodology employs polyvinyl alcohol (PVA) as a stimuli-responsive matrix, combined with polyaniline (PANI) serving as a functional component, while glutaraldehyde (GA) acts as the crosslinking agent. This PANI-based composite film overcomes the disadvantage that PANI does not easily form a uniform film. The maximum conductivity of this film can reach 0.034 S·cm^−1^. It is worth noting that the combination of PANI with the stimuli-responsive PVA film resulted in a composite film that not only retained good electrical conductivity, but also exhibited multiple stimuli-responsive properties. These stimuli-responsive properties can be controlled by external stimuli such as heat, voltage, light, or water. The PANI-based composite film could recover its original shape within 25 s when the applied voltage reached 30 V. These characteristics open up possibilities of potential applications where controlled deformation is desired.

## 1. Introduction

Functional materials are a class of advanced materials that possess unique properties and functionalities beyond those of conventional materials [1]. These materials play a crucial role in various fields including electronics, energy, healthcare, and environmental protection [2]. With the rapid advancement of science and technology, there is a growing demand for multifunctional materials that can meet the increasingly complex needs of modern society. These materials are not only required to possess excellent basic properties, but also need to integrate multiple functions to adapt to various application scenarios. For example, in the field of electronics, multifunctional materials are crucial for developing next-generation devices [3]. Traditional materials may only offer electrical conductivity or insulation properties, whereas new multifunctional materials can combine these properties with thermal management, flexibility, and even self-adaptive capabilities.

The development of electroactive polymers, an exciting field in materials science, can be traced back to a groundbreaking discovery in 1977 [4]. Alan J. Heeger, Alan G. MacDiarmid, and Hideki Shirakawa, who later received the Nobel Prize in Chemistry for their work, demonstrated that by introducing impurities into the polymer structure, its electrical properties could be dramatically altered [5,6,7,8]. This discovery was revolutionary because it challenged the conventional wisdom that polymers were typically insulators. Scientists began exploring other conductive polymers. The various electroactive polymers could change shape, size, or color in response to electrical stimuli [9]. These materials have since found applications in diverse fields such as electronics, sensors, actuators, and even artificial muscles.

Researchers are now investigating ways to improve the performance of these materials, aiming to unlock even more potential applications in the future. PANI is a conductive polymer that has garnered considerable attention due to its intriguing electrical and optical characteristics [10]. Moreover, the stability and durability of PANI under various environmental conditions contribute to its appeal. This robustness, combined with its low cost and ease of fabrication, positions PANI as a promising material for future technological advancements [11]. In practical applications, materials often need to withstand various forms of stress such as stretching, bending, or compression. However, PANI demonstrates relatively low mechanical strength [12]. Furthermore, PANI has poor solubility in common organic solvents [13]. Solubility is a critical factor in material processing, especially when it comes to creating uniform coatings or films. This limitation also complicates efforts to blend polyaniline with other materials to enhance its properties, thereby reducing its versatility. Usually, the rough surface of the PANI film can be beneficial for applications such as sensors, energy storage devices, and catalysis [12,13]. In sensor technology, the increased surface area provided by the roughness can lead to higher sensitivity and more effective interaction with the target molecules. In energy storage devices, such as supercapacitors or batteries, the rough surface can facilitate better electrode–electrolyte contact, improving the charge transfer efficiency and overall performance. The unique combination of surface characteristics and electrical conductivity makes the PANI film a promising candidate for advanced functional materials in multiple fields.

The functionalization and intelligentization of materials are gradually becoming the main trends of new material development [14,15,16]. This transition is driven by the increasing demand for materials that can not only adapt to various environments and perform multifunctional roles, but also exhibit intelligent responses to external stimuli. Shape memory polymers (SMPs) stand out as a typical example of intelligent materials, showcasing remarkable properties that make them highly valuable in numerous applications [17,18,19]. SMPs possess the unique ability to return to their original shape after being deformed when exposed to specific triggers such as heat, light, or magnetism [20,21,22]. This characteristic is achieved through the molecular structure of these polymers, which can be designed to have different phases or segments that respond differently to external conditions. For instance, some SMPs can be programmed to change shape at precise temperatures, making them ideal for use in adaptive structures, medical devices, and aerospace components. Moreover, the versatility of SMPs extends beyond their shape-changing capabilities. These materials can also be engineered to exhibit other desirable properties. In the medical field, SMPs are used in minimally invasive surgeries, where they can be inserted into the body in a compact form and then expanded to their original shape once inside, reducing trauma to surrounding tissues [23,24]. Similarly, in the automotive industry, SMPs can be incorporated into bumpers or panels that automatically repair themselves after minor impacts [25].

In recent years, PVA has garnered considerable attention in the development of smart materials [26]. PVA contains a large number of hydroxyl groups (–OH) and is a non-toxic, colorless, biodegradable, and water-soluble organic polymer with a high molecular weight [27,28]. The good hydrophilicity of PVA allows it to interact effectively with water, which is crucial for creating materials that respond dynamically to environmental changes [19]. Additionally, its tunability offers the flexibility to tailor its properties for specific uses. Its excellent hydrophilicity and tunability provide unique advantages in self-healing materials, water-responsive materials, and shape memory materials [29,30].

Traditional SMPs are thermal stimuli-responsive materials that have a high energy consumption and extremely difficult to contain [28]. So far, researchers have come up with a number of methods to overcome these disadvantages. This work presents a multifunctional stimuli-responsive polyaniline-based conductive composite film to overcome these shortcomings by utilizing PVA as a stimuli-responsive matrix, combined with PANI serving as functional component and GA acting as the crosslinking agent. The -OH of the PVA allows it to interact favorably with PANI, water, and other polar substances. This composite exhibits good film-forming properties, making it versatile in many applications. Therefore, PVA can form continuous and strong films with PANI. It also overcomes the disadvantage that PANI does not easily form a uniform film [31]. A series of experiments were conducted to explore the influences of various factors, including the PANI content, stirring time, and cross linking agent (GA) content, on the conductivity. One of the most remarkable features of the PANI-based composite film is its excellent shape memory function. In this case, the combination of PANI with the stimuli-responsive PVA results in a composite film that not only retains good electrical conductivity, but also possesses multiple stimuli-responsive capabilities. Researchers are exploring methods to enhance the performance of these materials, with the objective of unlocking a broader range of potential applications in the future.

## 2. Materials and Methods

### 2.1. Materials

PANI (98%), which exhibits a room temperature conductivity of 2 S·cm^−1^, was procured from Macklin Chemical Company (Shanghai, China). PVA and GA were purchased from Shanghai Aladdin Biochemical Technology Co., Ltd. (Shanghai, China). The acetic acid was obtained from Xilong Scientific (Shantou, Guangdong, China). All chemicals used in this experiment were used directly without additional treatment.

### 2.2. Characterization

The instruments used in this work included the following. Scanning electron microscopy (SEM, Hitachi S-4800, Hitachi, Ibaraki, Japan) was employed to examine the microscale surface morphology of the samples. Fourier transform infrared spectroscopy (FTIR) were acquired using a Perkin-Elmer spectrometer (Spectrum 100, Perkin-Elmer, Waltham, MA, USA).

Thermogravimetric analyses (TGA, TA Instruments Q600, New Castle, DE, USA) and differential scanning calorimetry (DSC, TA Instruments Model DSC Q100, New Castle, DE, USA) are widely used analytical techniques to study the thermal properties of materials. Firstly, a sample mass of 10 mg was selected for the TGA. The temperature ranges for this experiment spanned from room temperature up to 900 °C, providing a comprehensive view of the materials’ behavior across a wide thermal spectrum. The heating rate was set at 10 °C/min. To prevent unwanted reactions with oxygen or other gases in the environment, the test was conducted under a nitrogen (N_2_) atmosphere. A DSC instrument was employed to analyze the sample. The weight of the sample was approximately 5 mg. The temperature range for this DSC analysis was set from −10 °C to 250 °C. The heating and cooling rates were both maintained at 10 °C/min. To protect the sample from oxidation and other atmospheric influences, a N_2_ atmosphere was used. The flow rate of N_2_ was set at 30 mL/min, ensuring a steady and sufficient supply of inert gas throughout the experiment. The relative humidity was kept at 50%, and the ambient temperature was maintained at 25 °C. Here is an expanded explanation of each step in the process:

(1) Initial cooling phase: The sample is first cooled down from its initial temperature to −10 °C at a cooling rate of 10 °C/min. Once the temperature reaches −10 °C, it is held constant for 2 min.

(2) First heating: Following the initial cooling and soaking, the sample undergoes its first heating cycle. Starting from −10 °C, the temperature is increased at a consistent rate of 10 °C/min until it reaches 250 °C. After reaching 250 °C, the sample is maintained at this temperature for 2 min.

(3) Cooling phase: After the first heating cycle, the sample is cooled back down from 250 °C to −10 °C at the same cooling rate of 10 °C/min. Once again, after reaching −10 °C, the sample is kept at this temperature for 2 min to allow for complete thermal stabilization.

(4) Second heating: Following the cooling and holding phase, the sample undergoes a second heating cycle identical to the first one. It starts from −10 °C and is heated at a rate of 10 °C/min up to 250 °C. This heating rate is consistent with the previous cooling rate, suggesting a symmetrical thermal treatment. The temperature is raised until it reaches 250 °C.

Shape memory assessment is a critical process that evaluates the properties of shape memory materials. During the preliminary shape memory recovery experiments, we tested samples of several different temporary shapes such as spiral, triangular, or circular. These samples, with different temporary shapes, could all completely recover to their initial shape, verifying that the composite film has good shape memory properties. After comparing, we chose the “U” shape to evaluate the shape memory properties of the composite film in a more intuitive way. At the glass transition temperature (*T_g_*) + 20 °C, the straight specimen underwent a significant transformation, then was bent into a “U” shape. This process involved carefully applying heat to ensure that the material reached the required temperature uniformly. Once the desired “U” shape was achieved, the specimen was then cooled down to room temperature. During the cooling process, an external force was continuously applied to maintain the deformed shape, ensuring that the specimen retained its new configuration even as it cooled. Thermal-responsive shape recovery process was recorded with a digital camera at *T_g_* + 20 °C. Electrical-responsive and light-responsive shape recovery process were tested under an applied voltage of 30 V and the 300 mW/cm^2^ illumination condition, respectively. The water-responsive shape recovery performance was evaluated in water at room temperature. The temperature distribution was captured using a thermal infrared camera system (FOTRIC 225s, Shanghai, China). One end of the “U” shape sample was aligned at 0°, and the other end parallel to 0°. As the temperature increased to reach or exceed the *T_g_*, the polymer sample chains softened and began to move. The end parallel to 0° gradually recovered toward the 180° direction, allowing us to precisely determine the shape recovery ratio at any time through measurements. The formula for the shape recovery ratio is:

(1) $$R_{r}=\frac{\varepsilon_{r}}{\varepsilon_{o}}\times 100\%$$

where *R_r_* is the shape recovery ratio, $\varepsilon_{r}$ is the recovered angle during the shape memory recovery process, and $\varepsilon_{o}$ is the initial angle when the sample is in its original straight state. Herein, $\varepsilon_{o}$ is 180°.

A four-probe tester was used to test the conductivity (M-3 Mini type, Suzhou Jingge Electronic Co., Ltd., Suzhou, Jiangsu, China). The contact angle was measured using a contact angle goniometer (JC2000D4, POWEREACH, Shenzhen, Guangdong, China).

### 2.3. Fabrication of the Multifunctional Stimuli-Responsive Polyaniline-Based Conductive Composite Film

**Figure 1:**
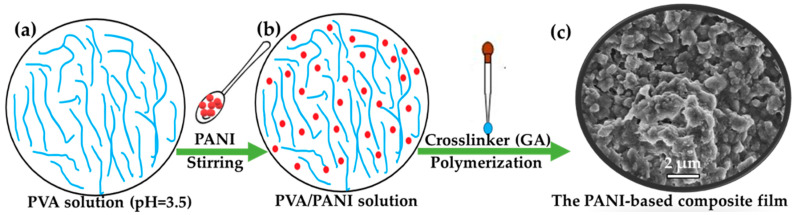
Schemes. Synthesis of the multifunctional stimuli-responsive polyaniline-based conductive composite film: (**a**) PVA solution; (**b**) PVA/PANI solution; (**c**) SEM image of the PANI-based composite film (m(PANI)/m(PVA) = 1.4, stirring time = 7 days, GA content = 0.09 vol%).

The consistency of the PVA water solution was maintained at 6 wt% with stirring at 100 °C until uniform dispersion (Figure 1a). This concentration was chosen to ensure the optimal solubility and stability of the PVA in solution. The pH of the PVA solution was adjusted to 3.5 using acetic acid. The PANI was mixed with the PVA solution under varying stirring times, in which the mass ratio of PANI to PVA (m(PANI)/m(PVA)) was 1.0, 1.1, 1.2, 1.3, 1.4, 1.5, and 1.6, as shown in Figure 1b (herein, the mass ratio of 1.0 means that the mass of PANI to PVA is 10:10; the mass ratio of 1.1 means that the mass of PANI to PVA is 11:10; the mass ratio of 1.2 means that the mass of PANI to PVA is 12:10; the mass ratio of 1.3 means that the mass of PANI to PVA is 13:10; the mass ratio of 1.4 means that the mass of PANI to PVA is 14:10; the mass ratio of 1.5 means that the mass of PANI to PVA is 15:10; the mass ratio of 1.6 means that the mass of PANI to PVA is 16:10). The stirring times in this step were 1, 2, 3, 4, 5, 6, 7, and 8 days, respectively. After adding the cross-linking agent (GA), the polymerization process was allowed to proceed for a duration of 2 h. This specific time frame was selected based on preliminary studies that indicated that it provided a sufficient reaction time for effective cross-linking without leading to excessive polymerization. Herein, the volume percentage (vol%) of GA in the total volume of the mixed solution was 0.05, 0.06, 0.07, 0.08, 0.09, 0.10, and 0.20, respectively. The entire process took place at room temperature. Throughout the experiment, continuous stirring was applied to ensure uniform mixing of the reactants. The mixture was utilized for the preparation of the films, then the film was allowed to dry naturally at room temperature. The films were washed with deionized water to remove residual PANI particles from their surfaces. Subsequently, the multifunctional stimuli-responsive polyaniline-based conductive composite films were dried naturally. Figure 1c shows the SEM image of the PANI-based composite film.

## 3. Results and Discussion

### 3.1. Microstructure and Chemical Properties

**Figure 2:**
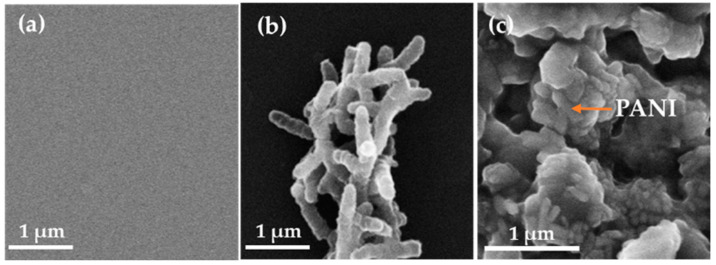
Microstructure. SEM images: (**a**) PVA film; (**b**) PANI powder; (**c**) PANI-based composite film (m(PANI)/m(PVA) = 1.4, stirring time = 7 days, GA content = 0.09 vol%).

Understanding the microstructure of materials is essential for optimizing their use in different fields. The surface microstructure of the samples was characterized using SEM. As shown in Figure 2a, the PVA film surface was flat and uniform [32]. The microstructure of the PANI powder exhibited a distinctive rod morphology (Figure 2b), which contributes significantly to the properties and potential applications of the materials [11]. These rods typically have a length ranging from 1 to 2 micrometers (μm) and a diameter of approximately 200 nanometers (nm). This rod shape provides a high aspect ratio, which can enhance the conductivity and mechanical strength of the composites. The length-to-diameter ratio affects how the PANI rods interact with other materials when incorporated into composite structures. Additionally, the uniformity of the PANI rods plays a crucial role in determining the overall performance of PANI-based devices or composites. The SEM image of the PANI-based composite film appeared to have a notably rough texture due to the introduction of PANI (Figure 1c). This roughness arose from the presence of a large amount of PANI, which was distributed relatively uniformly throughout the composite film, as shown in Figure 2c. The even distribution of PANI within the matrix led to the formation of conductive pathways or circuits, enhancing the electrical properties of the material.

**Figure 3:**
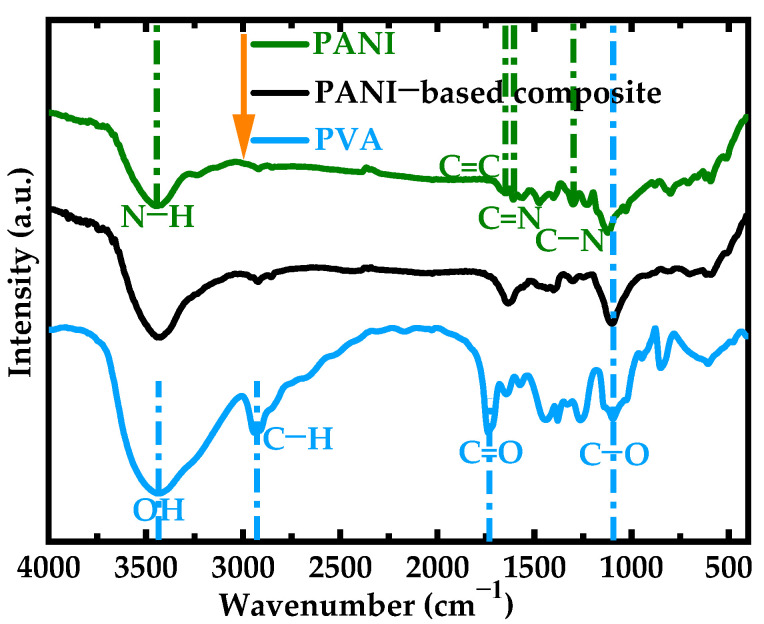
Chemical properties. FTIR spectra of the PVA film, PANI powder, and PANI-based composite film (m(PANI)/m(PVA) = 1.4, stirring time = 7 days, GA content = 0.09 vol%).

The infrared absorption spectra of the PVA film, the PANI powder, and the PANI-based composite film are illustrated in Figure 3. These spectra provide valuable insights into the molecular structures and interactions within these materials. For the PVA film, a prominent IR absorption peak was observed at 3439 cm^−1^, which can be attributed to the O–H stretching vibrations from the hydroxyl groups present in the PVA polymer chains [32]. This peak indicates the presence of hydrogen bonding between the hydroxyl groups, contributing to the stimuli-responsive and conductive properties of the film. One of the notable features in the infrared spectrum was the absorption peak at 1735 cm^−1^, which is typically associated with the C=O stretching vibration. This suggests that there may be partial acetylation or esterification occurring within the PVA molecule. The absorption peak at 1098 cm^−1^ was attributed to the C–O stretching vibration. This specific absorption band is a key indicator for identifying the presence of a carbon-oxygen (C–O) single bond in organic compounds.

In the case of the PANI powder, a peak appeared at 3445 cm^−1^ [33]. This peak corresponded to the N–H stretching vibrations from the amine groups in the PANI structure. The absorption peaks at 1607 cm^−1^ and 1296 cm^−1^ referred to C=N and C–N, respectively [34]. When examining the PANI-based composite film, it became evident that this material retained the characteristic peaks of both PVA and PANI. Specifically, the composite film exhibited peaks at 3435 cm^−1^, 1635 cm^−1^, and 1105 cm^−1^ [35]. These absorption peaks exhibited variations in their position and intensity due to differences in the molecular structure and environment as well as the chemical interactions between PANI, PVA, and GA. The slight difference in peak position compared with PVA suggests variations in the hydrogen bonding environment or the nature of the functional groups involved. The C–O appeared to be slightly lower than in PANI because of additional hydrogen bonding interactions. The presence of these groups suggests that the composite film incorporates various organic functionalities, enhancing its versatility for potential applications.

### 3.2. Thermodynamic and Thermal-Responsive Shape Memory Performance

**Figure 4:**
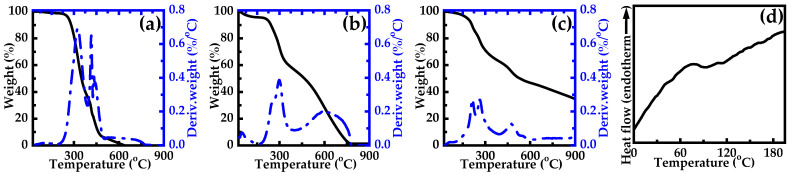
Thermodynamic performance. TGA thermograms of (**a**) the PVA film, (**b**) PANI powder, and (**c**) PANI-based composite film (m(PANI)/m(PVA) = 1.4, stirring time = 7 days, GA content = 0.09 vol%). (**d**) DSC thermograms of the PANI-based composite film (m(PANI)/m(PVA) = 1.4, stirring time = 7 days, GA content = 0.09 vol%).

The ability of the polymer to maintain its structural integrity and functional properties, when exposed to elevated temperatures, plays a crucial role in determining the performance of the materials and its applicability in various environments. Polymers with higher heat resistance can endure more extreme conditions without undergoing significant degradation or losing their intended functionalities. To evaluate the thermal stability of polymers, TGA is commonly employed. Figure 4a–c illustrates the thermal stability of the PVA film, PANI powder, and PANI-based composite film, respectively. PVA underwent thermal degradation at approximately 250 °C, while PANI degraded at around 210 °C. In contrast, the PANI-based composite film exhibited a slightly lower initial degradation temperature of about 195 °C. Specifically, for the PANI-based composite film, there was no significant weight loss observed until it reached its degradation temperature. The weight loss before reaching 350 °C was mainly due to the evaporation of physically adsorbed water molecules and other volatile components. After this initial phase, the rate of weight loss slowed down significantly compared with the PVA film and PANI powder. This behavior can be attributed to the interactions within the composite structure [12]. Remarkably, even at temperatures as high as 900 °C, the decomposition process was not complete, indicating the exceptional thermal stability of the PANI-based composite film.

Figure 4d shows the DSC thermogram of the PANI-based composite film. Specifically, its *T_g_* was approximately 80 °C [35,36]. This temperature is directly tied to the performance of the PANI-based composite film, indicating that the polymer chains within the material begin to lose their rigidity and its molecular segment activity is increased around this temperature [36]. These findings are essential for understanding the performance of the PANI-based composite film under different thermal conditions and to optimize its performance in practical applications.

At temperatures below the *T_g_*, the PANI-based composite film exhibited remarkable structural stability. Conversely, when the temperature rose above the *T_g_*, the PANI-based composite film underwent a significant transformation. The material became more malleable and adaptable, allowing its shape and structure to be adjusted with relative ease. One notable characteristic of this high-temperature state was the ability of the film to change from a straight configuration to a “U” shape. This shape-shifting property opens up possibilities for various applications where controlled deformation is desired. As the temperature gradually decreased back to below the *T_g_*, the PANI-based composite film could retain its newly acquired “U” shape. This shape memory effect is particularly valuable in fields such as smart materials and adaptive structures. The shape-fixing ability of the PANI-based composite film demonstrates its application potential for use in devices that require precise control over shape and function.

**Figure 5:**
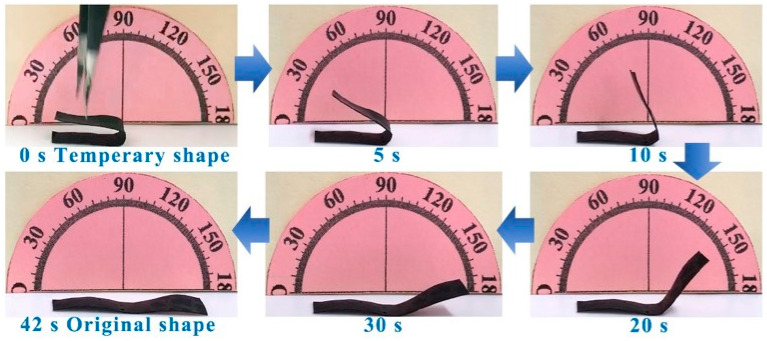
Thermal-responsive performance. Thermal-responsive shape recovery performance of the PANI-based composite film (m(PANI)/m(PVA) = 1.4, stirring time = 7 days, GA content = 0.09 vol%) at 100 °C (*T_g_* + 20 °C).

In Figure 5, the PANI-based composite film was placed on a heating plate at 100 °C (*T_g_* + 20 °C). As the temperature gradually increased, the PANI-based composite film started to undergo noticeable changes. This deformation was a result of the intrinsic properties of this PANI-based composite film, which responds dynamically to heat. The composite film continued to deform progressively until it eventually reached its original shape within 42 s. This recovery process was driven by the heat applied to the film [37]. This ability to return its original shape can be attributed to its shape memory properties, which are activated under specific temperature conditions. Such characteristics make this material promising for applications that require quick and reliable shape recovery in response to heat such as in smart textiles, deployable structures, or adaptive devices.

### 3.3. Electrical-Responsive Shape Memory Performance

The PANI-based composite films were prepared under various conditions to investigate the factors influencing their conductivity. The fixed conditions for the experiment were as follows. The consistency of the PVA water solution was maintained at 6 wt% with stirring at 100 °C until a uniform dispersion was reached. This concentration was chosen to ensure the optimal solubility and stability of the PVA in solution [27]. After adding the cross-linking agent (GA), the polymerization process was allowed to proceed for a duration of 2 h. This specific time frame was selected based on preliminary studies that indicated that it provided a sufficient reaction time for effective cross-linking without leading to excessive polymerization [27]. The entire process took place at room temperature. Throughout the experiment, continuous stirring was applied to ensure the uniform mixing of the reactants.

**Figure 6:**
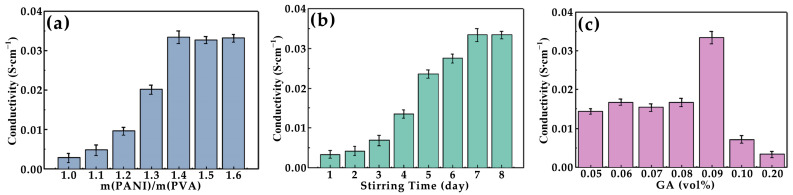
Conductivity. The impact of different factors on conductivity: (**a**) the mass ratio of PANI to PVA (stirring time = 7 days, GA content = 0.09 vol%); (**b**) stirring time (m(PANI)/m(PVA) = 1.4, GA content = 0.09 vol%); (**c**) cross linking agent (GA) content (m(PANI)/m(PVA) = 1.4, stirring time = 7 days).

The conductivity of these composite films was influenced by the distribution and density of PANI within the matrix. As the amount of PANI increased, it facilitated the formation of a more extensive and interconnected conductive network. This network allowed for better electron transfer and charge transport throughout the material. The PANI content not only directly impacts the number of conductive pathways, but also influences the overall structure and performance of the conductive network, thereby playing a key role in determining the conductive capability of the PANI-based composite films [12]. When the mass ratio of PANI to PVA reached 1.4 (14:10), the conductive capability of the PANI-based composite film was at its highest point. This optimal ratio indicates that there is a specific balance between PANI and PVA that maximizes the electrical conductivity of the resulting composite material. As shown in Figure 6a, further increasing the amount of PANI beyond this ratio did not significantly enhance the conductivity. The PVA serves as a matrix that supports the dispersion of PANI. When the mass ratio of PANI to PVA is too low, the conductive network formed by PANI is insufficient to achieve high conductivity. Conversely, when the ratio exceeds the optimal value, excess PANI may aggregate, leading to less effective charge transport pathways. The results suggest that achieving the right balance between these two components is crucial for optimizing the performance of the composite films [38]. For applications requiring conductivity, such as in electronic devices or sensors, this finding provides valuable insights into how to fine-tune the composition of the PANI-based composite films. Additionally, understanding the behavior of these materials at different ratios can help in designing composites with tailored electrical properties for various technological applications.

In the PANI-based composite films, the interaction between PANI and other components can affect the overall conductivity [12]. For example, if PANI is well-dispersed and forms strong interfacial bonds with other materials, it can significantly improve its conductive properties. The conductivity of the PANI-based composite films increased with the stirring time when the other conditions remained constant (Figure 6b m(PANI)/m(PVA) = 1.4, GA content = 0.09 vol%). This phenomenon can be attributed to the fact that prolonged agitation allows for a more uniform distribution of PANI within the PVA matrix. As the stirring time increases, the originally aggregated PANI particles are gradually dispersed throughout the PVA frame, leading to a more consistent and thorough integration of PANI within the composite structure. When the stirring time is insufficient, some areas of the PVA matrix may lack PANI or contain only minimal amounts, resulting in an uneven distribution and reduced overall conductivity. This non-uniformity creates discontinuities in the conductive pathways, which in turn lowers the overall electrical performance of the composite material. Through extensive experimentation, it was found that the optimal stirring time for achieving maximum conductivity was 7 days, at which point the conductivity reached its peak value of 0.034 S·cm^−1^. Beyond this point, further agitation did not contribute to any additional improvement in conductivity. This suggests that after 7 days, the PANI particles have already achieved an optimal dispersion within the PVA matrix, and any extra stirring time does not lead to a further enhancement of its properties [12]. The relationship between stirring time and conductivity highlights the importance of optimizing the processing parameters to achieve the best performance in the PANI-based composite films. Proper agitation ensures a homogeneous distribution of conductive components, thereby maximizing its electrical properties.

When the amount of cross-linking agent (GA) reached 0.09 vol% of the PVA solution, the conductivity of the PANI-based composite films peaked, as illustrated in Figure 6c. As the amount of GA increases, the polymerization process becomes more vigorous and intense. This heightened reactivity can lead to premature solidification of the mixed solution, which is undesirable for achieving optimal material properties [27]. The increased concentration of GA causes the PVA network to become denser. This densification has a direct impact on the PANI conductive network within the PANI-based composite films. Specifically, as the PVA network tightens, it refines and compresses the PANI conductive network, leading to a reduction in its overall conductive performance. Conversely, if the amount of GA is too low, it results in insufficient interaction between the components. This lack of adequate cross-linking creates gaps between the PANI chains, making the conductive network discontinuous and less dense. Such discontinuities significantly reduce the efficiency of electron transfer through the PANI-based composite films, thereby diminishing its conductive capabilities. Therefore, finding the appropriate amount of GA is crucial for constructing an optimal conductive network. An ideal balance ensures that the PVA network is sufficiently dense to provide structural integrity while allowing the PANI conductive network to remain continuous and well-distributed. This balance maximizes its conductive performance by ensuring efficient electron transport pathways throughout the PANI-based composite films. Additionally, the right proportion of GA can enhance the mechanical properties of the material. In practical applications, this means that the amount of cross-linking agent must be carefully controlled during the synthesis process.

These parameters are critical in determining the overall conductive performance of PANI-based composite films.

**Figure 7:**
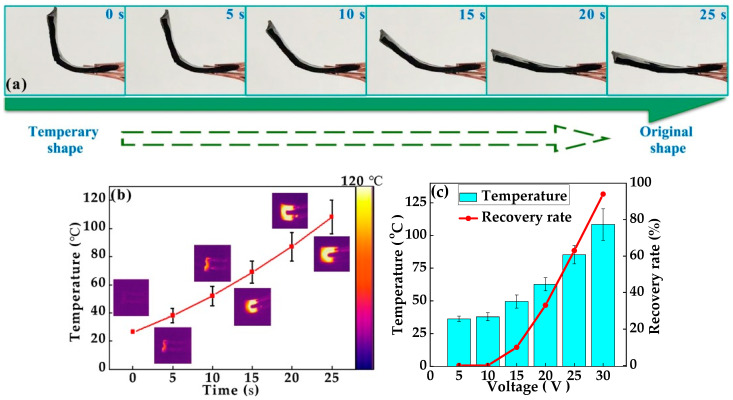
Electrical-responsive performance. Electrical-responsive shape recovery performance of the PANI-based composite film (m(PANI)/m(PVA) = 1.4, stirring time = 7 days, GA content = 0.09 vol%): (**a**) the shape memory recovery process was conducted using 30 V DC; (**b**) surface temperature change during shape memory recovery process and the embedded images are infrared thermal images; (**c**) surface temperature and shape memory recovery rate under different applied voltages.

When evaluating the electrical-responsive performance of the composite film, a direct current (DC) source was utilized for the testing procedure. The PANI-based composite film exhibited remarkable self-recovery properties when subjected to an electrical stimulus. Specifically, under an applied voltage of 30 V, the film was able to revert to its original state within just 25 s, as shown in Figure 7a. This rapid recovery can be attributed to Joule heating, which occurs due to an electric current passing through this conductive material [19]. The Joule heating causes the temporary cross-links to break once again. As the temperature rises above the *T_g_*, the polymer chains regain mobility. This increased mobility allows the PANI-based composite film to revert to its original permanent shape as the permanent cross-links guide the rearrangement of the polymer chains.

Understanding the exact timing and temperature dependencies can help optimize the performance of such materials. Figure 7b shows the surface temperature change during the shape memory recovery process. With the increase in the duration of voltage application, the temperature of the PANI-based composite film experienced a gradual rise. Initially, the temperature increase was subtle, but as time progressed, the thermal energy accumulated within the material [39]. After approximately 20 s of continuous voltage application, the temperature of the composite film could exceed its *T_g_*. The uniform temperature distribution across the surface of the PANI-based composite film is particularly noteworthy and suggests that the heat generated by the applied voltage was evenly dispersed throughout the material, without any localized hotspots. Such uniformity is crucial for maintaining the structural integrity and performance consistency of the film during operation.

In Figure 7c, it is evident that when the applied voltage was lower than 10 V, the PANI-based composite film remained at a relatively low temperature. Stability at low voltages is crucial for applications where consistent performance without unintended deformation is required [39]. As the voltage increased beyond 10 V, the material began to heat up, and this rise in temperature enabled the shape memory effect to activate. The extent of this activation varies depending on the maximum temperature reached. Specifically, higher voltages lead to higher temperatures, thereby enhancing the ability to recover its original shape. When the voltage reached 30 V, the material demonstrated a high shape memory recovery rate, effectively returning to its original configuration within 25 s. Moreover, the relationship between the voltage and temperature in this material highlights the importance of thermal management in optimizing the shape memory performance. Such rapid and reliable recovery could be utilized in developing self-healing coatings or actuators that respond dynamically to external stimuli.

### 3.4. Light-Responsive Shape Memory Performance

**Figure 8:**
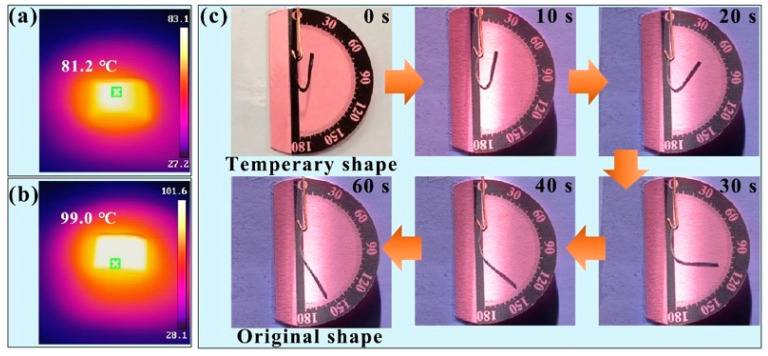
Light-responsive performance. The infrared thermal image of the PANI-based composite film (m(PANI)/m(PVA) = 1.4, stirring time = 7 days, GA content = 0.09 vol%) acquired at a light intensity of (**a**) 100 mW/cm^2^ and (**b**) 300 mW/cm^2^; (**c**) the light-responsive shape recovery process was conducted at an irradiance of 300 mW/cm^2^.

To record the temperature variations more accurately, an infrared thermal imaging technique was employed, as shown in Figure 8a,b. When exposed to light with intensities of 100 mW/cm^2^ and 300 mW/cm^2^ for a duration of 2 min, the sample demonstrated significant temperature increases, reaching 81.2 °C and 99 °C, respectively. The rate of the temperature increases is directly influenced by the light irradiation intensity. Higher light irradiation intensity leads to a faster heating rate and ultimately results in a higher peak temperature. For instance, under the 300 mW/cm^2^ condition, the sample not only heated up more rapidly, but also attained a higher maximum temperature compared with the 100 mW/cm^2^ condition.

As shown in Figure 8c, the light-responsive shape memory effect of the PANI-based composite film was measured with a fixed light intensity of 300 mW/cm^2^. This light intensity was chosen because it is a suitable level for triggering the desired response without causing damage or overheating the composite material. It was observed that the “U” shape of the PANI-based composite film underwent significant changes after 10 s, with the deformation becoming increasingly pronounced over time. This facilitates the utilization of the PANI-based composite film as light-responsive SMP system. In this system, PANI acts as an effective light-absorbing filler, efficiently converting light energy into thermal energy [40]. As a result, this composite film can respond to light stimuli by altering its shape and recovering its original form. The composite materials exhibit the characteristic of rapid response to light irradiation. In aerospace, light responsive SMPs can be used to create adaptive wing structures. Moreover, in space equipment such as satellite antenna, it can also use its characteristics to achieve compact storage and expansion functions. In daily life, light-responsive SMPs also have many applications. For example, in smart homes, they can be used to make curtains that automatically adjust the opening and closing degree according to the ambient light. In clothing design, new fabrics that change styles or patterns with sunlight can be developed to bring people a more personalized and intelligent life experience.

### 3.5. Water-Responsive Shape Memory Performance

**Figure 9:**
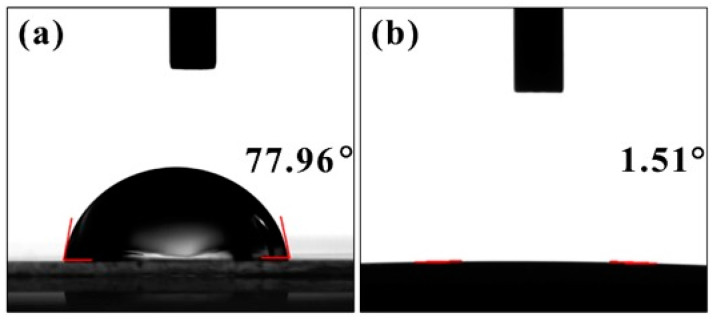
Hydrophilic performance. The water contact angel test photos of (**a**) the PVA film and (**b**) PANI-based composite film (m(PANI)/m(PVA) = 1.4, stirring time = 7 days, GA content = 0.09 vol%). The photos were taken at 2 s after the samples were touched with a water drop.

PVA films are known for their hydrophilic properties due to the presence of a large number of hydrophilic groups on their surface [27], as illustrated in Figure 3 and Figure 9a. Despite this characteristic, this PVA film exhibited limited water absorption because of its dense surface structure, as shown in Figure 2a. The dense arrangement of molecules restricted the penetration of water molecules into the film, thereby limiting its overall water absorption capacity. In contrast, the PANI-based composite films possessed not only the hydrophilic groups from both PVA and PANI, as depicted in Figure 3, but also an irregular microstructure that significantly enhanced its specific surface area, as seen in Figure 2c. This unique microstructure created more contact points between the film and water molecules, leading to increased interaction and better wettability. Consequently, the PANI-based composite film demonstrated superior hydrophilicity compared with the PVA film alone. The enhanced hydrophilicity of the PANI-based composite film opens up possibilities for its application in liquid environments.

**Figure 10:**
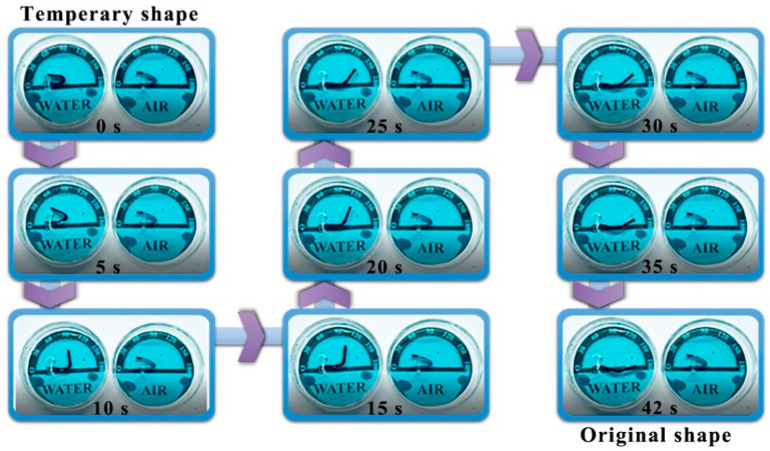
Water-responsive performance. Water-responsive shape recovery performance of the PANI-based composite film (m(PANI)/m(PVA) = 1.4, stirring time = 7 days, GA content = 0.09 vol%) at room temperature.

The deformed sample was carefully immersed in water at room temperature, and the changes in its shape were recorded over time using a digital camera, as shown in Figure 10. This experimental setup allowed for documentation of the shape memory process. The PANI-based composite film, when placed in water, exhibited a remarkable recovery to its initial state within just 42 s. In contrast, when the same type of composite film was left in air, it remained unchanged, demonstrating the stability of the material in an atmospheric environment. These observations highlight the controllability of shape adjustment in the PANI-based composite films, which can be triggered by various stimuli. While thermal, electric, and light-driven mechanisms have been previously explored, this study also introduced water as an additional and effective driving force. The mechanism behind the rapid shape recovery of the water-induced PANI-based composite film can be attributed to the penetration of water molecules into the PVA matrix. These water molecules act as plasticizers, facilitating the rearrangement of polymer chains and enabling the material to regain its original form [27]. This process underscores the importance of molecular interactions within the composite structure and provides insights into how external factors can modulate the material properties. Furthermore, the stability of the PANI-based composite film in air suggests that the material can maintain its integrity under normal environmental conditions, making it suitable for practical applications where exposure to air is inevitable. The combination of stability in air and responsiveness to water offers a versatile platform for developing smart materials with tailored functionalities.

## 4. Conclusions

In summary, our multifunctional stimuli-responsive polyaniline-based conductive composite film not only demonstrates good basic properties, but also integrates multiple functions to adapt to multiple application scenarios. The composite films, which demonstrated good film-forming characteristics, were prepared using different ratios of PANI, PVA, and GA. Such investigations help in tailoring preparation methods to achieve the desired morphologies and properties, thereby expanding the range of potential applications for PANI materials. The uniformly distributed PANI rods is an important factor in determining the overall performance of the PANI-based composite film, whose maximum conductivity could reach 0.034 S·cm^−1^. The PANI-based composite film could also recover to its original state within 25 s under an applied voltage of 30 V. Furthermore, it also exhibited a notable capability to respond to light stimuli, as PANI serves as an efficient light-absorbing filler, converting light energy into thermal energy with remarkable efficiency. In the PANI-based composite film, the hydroxyl groups of the PVA matrix contributed to favorable interactions with PANI, water, and other polar substances. Hence, the composite film combines the advantages of PVA and PANI, which benefits the interaction with water molecules for increasing plasticization with the rearrangement of polymer chains, so that it recovered quickly to its initial state within just 42 s in water at room temperature. These characteristics make this material promising for advanced technological applications that demand high adaptability and responsiveness such as adaptive devices, smart textiles, deployable structures, or sensor technology.

## Data Availability

Data are contained within the article.
